# Pulsed electromagnetic fields combined with a collagenous scaffold and bone marrow concentrate enhance osteochondral regeneration: an *in vivo* study

**DOI:** 10.1186/s12891-015-0683-2

**Published:** 2015-09-02

**Authors:** Francesca Veronesi, Matteo Cadossi, Gianluca Giavaresi, Lucia Martini, Stefania Setti, Roberto Buda, Sandro Giannini, Milena Fini

**Affiliations:** Department Rizzoli RIT, Laboratory of Biocompatibility Innovative Technologies and Advanced Therapies, Via Di Barbiano 1/10, 40136 Bologna, Italy; I Orthopaedics and Trauma Clinic, Rizzoli Orthopaedic Institute, Via Di Barbiano 1/10, 40136 Bologna, Italy; University of Bologna, Bologna, Italy; Laboratory of Preclinical and Surgical Studies, Rizzoli Orthopaedic Institute, Via Di Barbiano 1/10, 40136 Bologna, Italy; IGEA S.p.A., via Parmenide 10/A, 41012 Carpi, Modena Italy

## Abstract

**Background:**

The study aimed to evaluate the combined effect of Pulsed Electromagnetic Field (PEMF) biophysical stimulation and bone marrow concentrate (BMC) in osteochondral defect healing in comparison to the treatment with scaffold alone.

**Methods:**

An osteochondral lesion of both knees was performed in ten rabbits. One was treated with a collagen scaffold alone and the other with scaffold seeded with BMC. Half of the animals were stimulated by PEMFs (75 Hz, 1.5 mT, 4 h/day) and at 40 d, macroscopic, histological and histomorphometric analyses were performed to evaluate osteochondral defect regeneration.

**Results:**

Regarding cartilage, the addition of BMC to the scaffold improved cell parameters and the PEMF stimulation improved both cell and matrix parameters compared with scaffold alone. The combination of BMC and PEMFs further improved osteochondral regeneration: there was an improvement in macroscopic, cartilage cellularity and matrix parameters and a reduction in the percentage of cartilage under the tidemark. Epiphyseal bone healing improved in all the osteochondral defects regardless of treatment, although PEMFs alone did not significantly improve the reconstruction of subchondral bone in comparison to treatment with scaffold alone.

**Conclusions:**

Results show that BMC and PEMFs might have a separate effect on osteochondral regeneration, but it seems that they have a greater effect when used together. Biophysical stimulation is a non-invasive therapy, free from side effects and should be started soon after BMC transplantation to increase the quality of the regenerated tissue. However, because this is the first explorative study on the combination of a biological and a biophysical treatment for osteochondral regeneration, future preclinical and clinical research should be focused on this topic to explore mechanisms of action and the correct clinical translation.

## Background

Regenerative therapy for osteochondral lesions aims to produce a durable cartilage-like and bone tissues with the same structure and function as the native cartilage and well integrated with the surrounding tissues [1]. However, until now, the traditional surgical strategies have not been able to repair completely osteochondral lesions, but often produce a fibrous or fibrocartilaginous tissue that undergoes degeneration in the long term, with reduced biological, biomechanical and biochemical features [2].

Recently, tissue engineering (TE) approaches have emerged in the scenario of orthopedics as a promising alternative to overcome the limitations of traditional surgery [3]. Among the different scaffolds, collagen-based ones provide a natural cartilage and bone microenvironment, which promote mesenchymal stem cell (MSC) or chondrocyte attachment, proliferation, activity and extracellular matrix (ECM) deposition [4].

A one-step TE procedure, based on the use of bone marrow concentrate (BMC) transplantation, has gained popularity because it overcomes the limits and risks of the *in vitro* MSC expansion procedures (long times, costs, cell transformation, contamination and unnatural differentiation), usually performed to obtain a useful amount of cells for scaffold colonization. BM cells can be harvested easily from the patient’s iliac crest, concentrated directly in the operating theatre and implanted arthroscopically once seeded onto a scaffold, thus avoiding the need for two surgical stages (one for cell harvesting and another for implantation) [4]. In addition, autologous BMC carries also accessory cells and several growth factors that induce angiogenesis and vasculogenesis [5].

Hyaluronic acid and collagen have been used for osteochondral regeneration in combination with BMC for the regeneration of osteochondral lesions in patients. Good results have been obtained independently of the scaffold used in some cases [5, 6], whereas better results have been reported with a collagen scaffold compared to a hyaluronic acid membrane in others [7].

There is an increasing awareness of the importance of the microenvironment where both scaffolds and cells are transplanted. The inflammatory joint microenvironment, produced by the lesion itself or subsequent to the surgical procedure, should be considered as an important variable that causes MSC differentiation towards a fibroblastic phenotype and might also affect scaffold degradation [8]. The rationale for using Pulsed Electromagnetic Fields (PEMFs) in association with TE strategies was recently supported by evidence of both an anabolic effect on implanted cells and surrounding tissues and an anti-inflammatory effect protecting cells from the catabolic effects of inflammation [8]. Besides improvements in cartilaginous tissue healing [8, 9], it has been observed that biophysical stimulation with PEMFs, promotes osteochondral graft healing and integration [10, 11]. Boopalan *et al*., showed the effectiveness of PEMF stimulation in hyaline cartilage formation six weeks after the implantation of a calcium phosphate scaffold in ostechondral defects in rabbits [12].

Therefore, the hypothesis of the present *in vivo* study was that the combination of PEMF stimulation and the seeding of BMCs onto a collagenous scaffold might have a potential additive or synergic therapeutic effect in enhancing osteochondral regeneration in comparison to scaffold alone.

We evaluated the regenerative potential through macroscopic, histological and histomorphometric analyses in a rabbit model of osteochondral defect.

## Methods

The study was performed according to European and Italian legislation on animal experimentation (Law by Decree No.116/92). The experimental protocol was approved by the Ethical Committee of Rizzoli Orthopaedic Institute and Italian Ministry of Health. Ten adult male New Zealand rabbits (about 5 months old, 3.0 ± 0.3 kg) (HARLAN Laboratories SRL, Udine-Italy) were housed under controlled conditions and supplied with standard diets. General anaesthesia was induced with an intramuscular injection of 44 mg/kg ketamine (Imalgene 1000, Merial Italia SPA, Padova-Italy) and 3 mg/kg xylazine (Rompum, Bayer Italia SpA, Milan-Italy) and maintained with O_2_ and air (0.5 l/min) mixed with 2-3 % Isofluoran (Aerrane, Baxter S.p.A, Rome-Italy) in spontaneous ventilation.

### BMC isolation

Before joint surgery, 6.0 ± 1.5 ml of BM from the posterior iliac crest of each animal was aspirated into a syringe coated with saline-heparin solution and the needle was rotated to prevent venous return. The samples were immediately sent to the laboratory and an equal volume of physiological solution (Fresenius Kabi Italia s.r.l., Verona-Italy) was added to the BM aspirate, then stratified on Ficoll-Paque (density 1.083 g/ml) (Sigma-Aldrich, Milan-Italy) and subsequently centrifuged at 600 g for 30 min. The low-density cellular layer was separated, counted and resuspended in 200 μl of physiological solution for immediate surgical implantation.

### Joint surgery

Through a lateral knee arthrotomy, an osteochondral defect of 4 × 4 mm was performed in the loading area of both medial femoral condyles with a dimension comparable to that of a clinical microfracture [13]. In each animal, one defect was filled with a scaffold (BIOPAD, Novagenit, Trento-Italy) consisting of heterologous equine type I collagen, and the other one with the scaffold seeded with BMC (2.03 × 10^6^ BM mononuclear cells). The joint capsule and skin were sutured and after surgery the animals received antibiotics, 30 mg/Kg of Flumequine (Flumexil, Fatro SpA, Bologna-Italy) for 4 d and analgesics, 80 mg/Kg of sodium metamizole (Farmolisina, Ceva SpA, Monza-Italy).

From the first post-operative day, half of the animals underwent biophysical PEMF stimulation with 1.5 mT, 75 Hz (I-ONE, Igea SpA, Modena-Italy) 4 h/day until the end of the experimental time. Two solenoids, positioned at the level of articulations, were placed outside Plexiglas cages and connected to a pulsed generator. The same conditions were maintained also in the other half of the animals, but without the activation of the generator (not stimulated animals).

After 40 d, under general anaesthesia, the animals were pharmacologically euthanized with intravenous injection of 1 ml Tanax (Hoechst AG, Frankfurt-am-Mein-Germany).

Four groups of five treated knees were examined: Group 1 (scaffold), Group 2 (scaffold seeded with BMC), Group 3 (scaffold and PEMFs), and Group 4 (scaffold seeded with BMC and PEMFs).

### Post-surgical evaluations

The Niederauer score was used for gross morphological evaluation [14].

Both condyles of all animals were processed and sectioned as in a previous study [15]. For each sample three consecutive central sections of the entire volume of the lesion were stained with Safranin-O/Fast Green. Histological analysis was performed by a light optical microscope (Olympus-BX51, Italia Srl, Milan-Italy), equipped with a camera and connected to an imaging analysis system (Leica QWIN, Leica Microsystems Srl, Milan-Italy). A semi-quantitative O’Driscoll modified grading score [16] was used to evaluate both cartilage and bone compartments by two blinded investigators (Table 1).

**Table 1:** Scoring parameters for histological evaluations (modified O’Driscoll score)

Tissue	Parameters	Score				
		0	1	2	3	4
Cartilage (Min-Max: 0–31)	Tissue morphology	Fibrous tissue	Mostly fibrocartilage	Mix Hyaline cartilage and fibrocartilage	Mostly hyaline cartilage	Hyaline cartilage
	Matrix staining	None	Slight	Moderate	Normal	
	Surface regularity	Disrupted surface	Fissuring	Horizontal lamination of surface	Smooth and intact	
	Structure integrity	Severe disintegration	Slight break	Normal		
	Neo-cartilage alignment with native cartilage	Depressed	Elevated	Leveled		
	Thickness of neo-cartilage	No cartilage	50-100 % of normal cartilage	Similar to normal		
	Bonding to the native cartilage	Not bonded	Partially bonded at both ends or bonded at one end	Bonded at the both ends		
	Chondrocyte clustering	25-100 % of the cells	<25 % of cells is grouped in clusters	No clusters		
	Hypocellularity	Severe	Moderate	Slight	Normal	
	Cell distribution	Disorganized	Moderate disorganization	Slight disorganization	Columnar	
	Degenerative changes in the adjacent native cartilage	Severe hypocellularity, poor or no staining	Mild or moderate hypocellularity, slight staining	Normal cellularity, mild clusters, moderate staining	Normal cellularity, no clusters, normal staining	
	Tidemark continuity	Absent	Partial	Present		
Bone (Min-Max: 0–12)	Reconstruction of subchondral bone	No reconstruction	Minimal	Reduced	Normal	
	Bone infiltration into defect area	None	Partial	Complete		
	Bonding with adjacent bone	Without continuity on either edge	Partial on both edges	Complete on one edge	Complete on both edges	
	Subchondral bone morphology	Only fibrous tissue	Compact bone and fibrous tissue	Compact bone	Trabecular with some compact bone	Normal trabecular bone

The amount of new cartilaginous tissue, over and under the tidemark, was calculated in a region of interest (ROI) inside the osteochondral defect [17]. The percentage (%) of new cartilage, obtained by binarizing the area of positive Safranin-O staining, was calculated as (new cartilage area/ROI area) × 100.

### Statistical analysis

Statistical analysis was performed using the IBM SPSS Statistics v.21 software. After checking the normal distribution and the homogeneity of the variance, the non-parametric Kruskal-Wallis test was used to verify if there were significant differences in the macroscopic and histomorphometric results among groups. Then, the Mann–Whitney U test was evaluated by the Monte Carlo method to compute two-sided probability to compare the results of Group 2, Group 3 and Group 4 with Group 1.

## Results

### Macroscopic evaluations

No surgical or post-operative complications were observed and the animals tolerated both surgery and PEMF stimulation well. The Group 1 defects were partially empty with a rough brown fibrous tissue surface and a slight depression in the center and those of Group 2 appeared translucent with a high degree of filling and integration. In both cases the margins of the lesion were almost indistinguishable. Group 3 presented irregularities in the well-integrated and barely noticeable surface that was approximately filled up to the level of adjacent cartilage and those of Group 4 were filled with a fully-integrated and transparent cartilage-like tissue, quite indistinguishable from normal adjacent tissue.

**Fig. 1:**
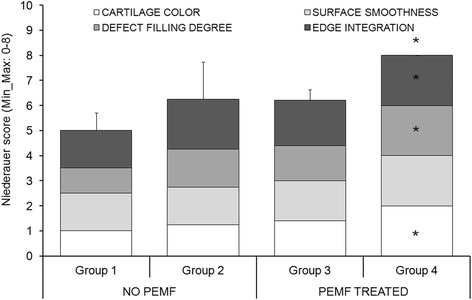
Niederauer score results of macroscopic evaluations (Mean ± SD, *n* = 5). Mann–Whitney U test (*p* < 0.05): *, Group 4 *versus* Group 1

The total and subcategories Niederauer score showed significantly higher values in Group 4 in comparison with Group 1 (*p* < 0.05), except for the surface smoothness parameter (Fig. 1).

### Histological and histomorphometric evaluations

Forty days after surgery, the scaffold was no longer recognizable in any of the Groups and no inflammatory cells or fibrous capsules were found regardless of treatment.

**Fig. 2:**
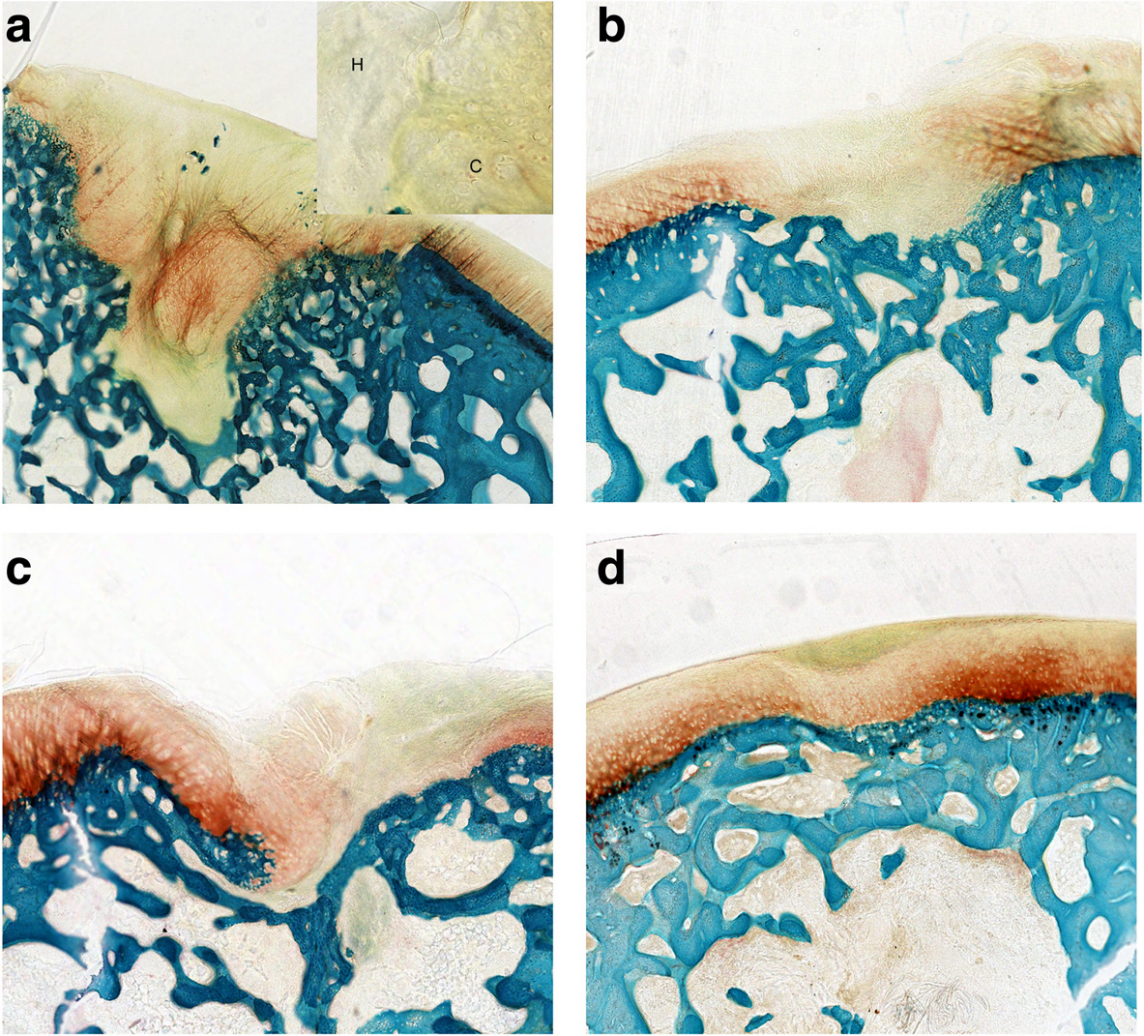
Histological images of the osteochondral lesions: **a** Group 1 – scaffold; **b** Group 2 – scaffold seeded with BMC; **c** Group 3 – scaffold and PEMFs; **d** Group 4 –scaffold seeded with BMC and PEMFs. H = Hypocellularity; C = Chondrocyte cluster. Safranin-O/Fast Green staining. Magnification of 4X

The Group 1 defects were characterized by the presence of a fibrocartilage, with a poor glycosaminoglycan (GAG) staining, an altered distribution of chondrocytes, grouped in clusters, and zones of hypocellularity (Fig. 2a). Group 2 showed a fibrocartilage with a normal cell distribution, few clusters and slight hypocellularity (Fig. 2b). After PEMF stimulation, the repaired tissue of Group 3 was a mix of hyaline cartilage and fibrocartilage with moderate matrix staining, slight fibrillation and some clusters of chondrocytes in a few zones (Fig. 2c). Group 4 defects presented mainly hyaline cartilage, with a normal GAG content, nearly smooth surface, no clusters, no hypocellularity and cells with a columnar aspect (Fig. 2d). In all Groups, both edges were integrated with the adjacent cartilage, which appeared normal with no signs of degeneration. In Group 4 bone reconstruction was complete.

**Fig. 3:**
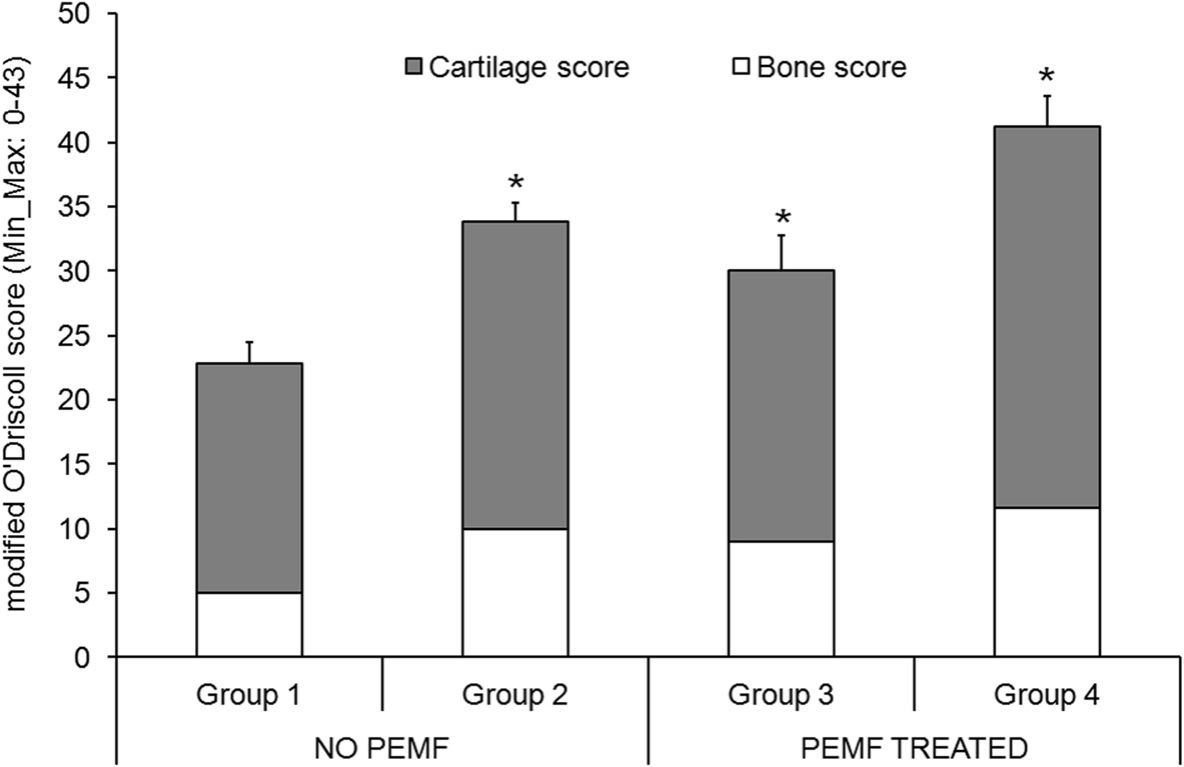
Modified O’Driscoll score results of cartilage and bone tissues (Mean ± SD, *n* = 5). Mann–Whitney U test (*p* < 0.05): *, all Groups *versus* Group 1

Table 2 and Fig. 3 show the modified O’Driscoll score results.

**Table 2:** Results of each parameter of modified O’Driscoll score (Mean, *n* = 5)

Tissue	Parameters	No PEMF		PEMF treated	
		Group 1	Group 2	Group 3	Group 4
Cartilage	Tissue morphology	1.4	1.6	2.6^c^	2.6
	Matrix staining	1.2	1.8	1.8	3.4^e^
	Surface regularity	2.2	2.6	2.4	2.6
	Structure integrity	1.4	1.6	1.6	2.0
	Neocartilage alignment	0.8	1.6	1.2	2.4^e^
	Chondrocyte clustering	1.8	2.0	1.2	2.0
	Hypocellularity	0.8	2.0^b^	2.0^d^	3.4^f^
	Degenerative changes in adjacent cartilage	2.8	3.0	2.8	3.0
	Bonding to native cartilage	2.0	2.0	2.0	2.0
	Cell distribution	0.6	2.6^a^	1.0	3.4^f^
	Thickness of neocartilage	1.8	2.0	1.6	2.0
	Tidemark continuity	1.0	1.0	0.8	1.0
Bone	Reconstruction of subchondral bone	1.2	2.6^a^	1.4	3.0^f^
	Bone infiltration	1.0	2.0^b^	1.6^c^	2.0^f^
	Bonding with adjacent bone	1.2	2.8^a^	2.8^c^	3.0^f^
	Subchondral bone morphology	1.6	2.6^a^	3.2^c^	3.6^f^

Mann–Whitney U test: ^a^, Group 2 *versus* Group 1 (*p* < 0.05); ^b^, Group 2 *versus* Group 1 (*p* < 0.005); ^c^, Group 3 *versus* Group 1 (*p* < 0.05); ^d^, Group 3 *versus* Group 1 (*p* < 0.005); ^e^, Group 4 *versus* Group 1 (*p* < 0.05); ^f^, Group 4 *versus* Group 1 (*p* < 0.005)

Considering the effect of BMC, the total modified O’Driscoll score was significantly higher in Group 2 than in Group 1 (*p* < 0.05), with an improvement in hypocellularity (*p* < 0.005), cell distribution (*p* < 0.05), bone infiltration (p < 0.005) and the other bone parameters (*p* < 0.05).

Regarding PEMF effect, the total modified O’Driscoll score was significantly higher in Group 3 than in Group 1 (*p* < 0.05), with low hypocellularity (*p* < 0.005) and better tissue morphology (*p* < 0.05) and bone microarchitecture parameters (*p* < 0.005), except for the reconstruction of bone parameters.

Group 4 showed a significantly higher modified O’ Driscoll score (*p* < 0.05) than Group 1, concerning matrix staining, neocartilage alignment (*p* < 0.05), hypocellularity and cell distribution (*p* < 0.005) and bone parameters (*p* < 0.005).

**Fig. 4:**
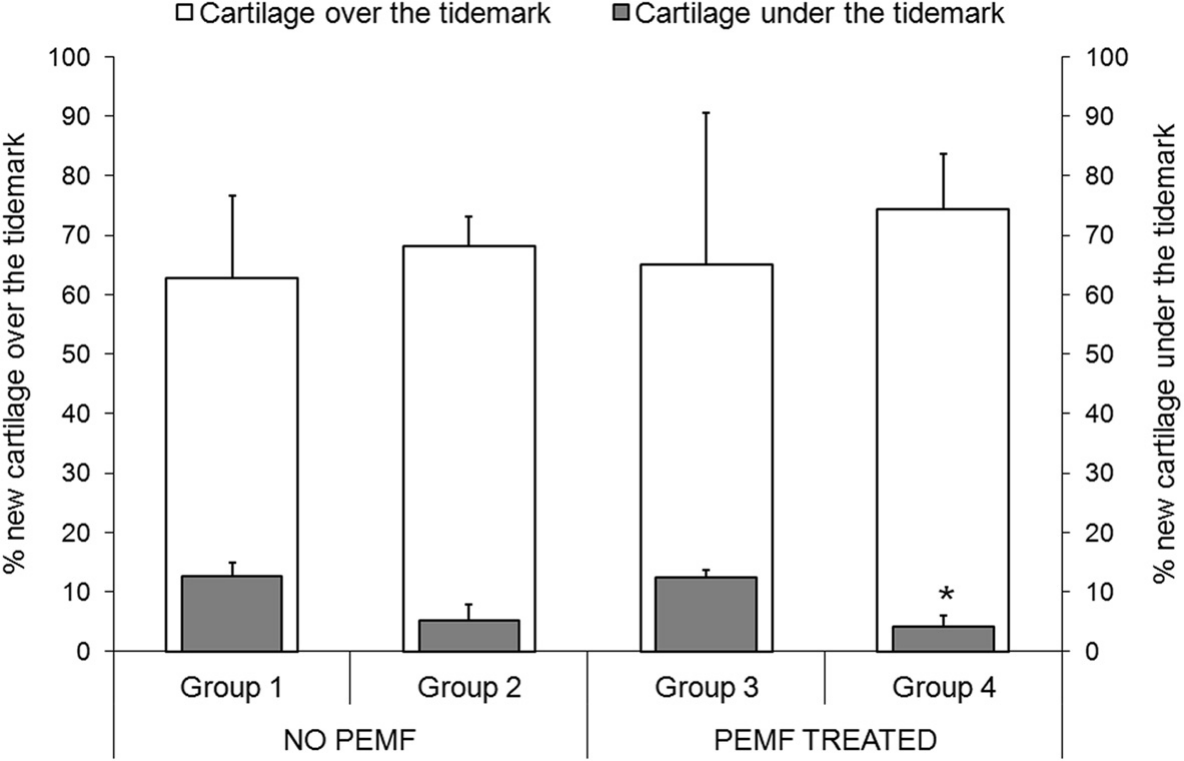
Percentage of new cartilage growth over and under the tidemark (Mean ± SD, *n* = 5). Mann–Whitney U test (*p* < 0.05): *, (new cartilage growth under the tidemark) Group 4 *versus* Group 1

Finally, the percentage of cartilage under the tidemark was significantly lower in Group 4 than in Group 1 (*p* < 0.05) (Fig. 4).

## Discussion

This study evaluated the separate and combined effect of BMC implantation and PEMF stimulation on osteochondral healing potential of collagenous scaffold alone.

Besides cartilage, the bone compartment was also assessed because proper bone healing influences the long-term articular cartilage repair, with high quality and smoother surface, gives mechanical support for both cartilage formation and lateral integration and protects cartilage from loss and stresses [18].

Seeding BMC on the scaffold improved the cartilage cellularity parameters of the modified O’Driscoll score, because BM cells are able to undergo a chondrogenic differentiation once implanted in the joint microenvironment and influence the microenvironment through a paracrine effect. Similar results were also observed in previous studies where enhanced healing of equine and mini-pig osteochondral defects was obtained with BMC [19, 20].

PEMF stimulation significantly improved not only cartilage cellularity, but also the neo-tissue morphology, whereas the combination of PEMFs and BMC improved their separate effects: macroscopic appearance, cellularity, neo-tissue staining and alignment were significantly better and the amount of cartilage under the tidemark was significantly reduced when PEMFs were combined with BMC in comparison to scaffold alone. The amount of cartilage under the tidemark is an index of subchondral bone regeneration, because the less the subchondral bone defect is filled by cartilage or fibrous tissue, the more it is filled by bone [17]. The histological parameters of bone improved, regardless of treatments, even if only the reconstruction of subchondral bone in Group 3 was similar to that of Group 1. In agreement with literature data [18], in this study the improvement of bone was associated not only with a better trabecular bone architecture, but also an improvement in cartilage cellularity, structure, alignment and ECM component synthesis, thus confirming that a proper bone regeneration contributes to a better cartilage repair.

It is also important to underline that lateral integration of neocartilage and attachment to the underlying bone occurred in all the treated groups.

In the current study a scaffold already used with good results in clinical practice was implanted, to measure the effect of BMC and PEMFs and to avoid the variability of innovative scaffolds and to easily translate our research results to patients. Forty days after surgery, the scaffold was no longer present in any treatment group but was replaced by more or less mature cartilage and bone tissues.

In the treatment of osteochondral defects several studies have evaluated separately the biophysical stimulation [10, 12] and BMC implantation technique [4, 19, 20] but to our knowledge, there is a lack of preclinical data on the combined effects of the cell therapy and PEMFs on osteochondral defect regeneration. In addition, a previous *in vitro* study by Wang Q et al. also underlined the effects of PEMF on MSC osteogenic differentiation as a new TE technique to be employed before the *in vivo* use of MSC for bone regeneration [21]. The authors observed the osteogenic differentiation of amniotic epithelial cells, as the MSC source, thus showing that the combination of PEMF stimulation and osteoinductive medium improved cell osteogenic differentiation in comparison to their use separately [21]. In the scope of a larger project it was recently shown by Cadossi *et al.* that PEMF stimulation improved recovery, pain control and clinical outcome in patients treated with collagen scaffold combined with BMC in osteochondral lesions of the talus after 60 d of treatment [22]. The present *in vivo* data strongly support the first clinical evidence with the help of histological and histomorphometric analyses, but the short-term follow-up and the single experimental time do not allow further maturation or gradual deterioration of the regenerated tissues to be observed. In addition, a mechanistic explanation of the PEMF effects has to be explored in other specific studies with an appropriate set-up.

## Conclusions

The results suggest that a TE approach, represented by BMC seeding on a collagen scaffold, combined with low-frequency PEMF stimulation, rather than separately, improves osteochondral regeneration, thus confirming the hypothesis of the study. Even if all treatments significantly enhanced bone architecture, only the combined use of BMC and PEMF stimulation improved cartilage cellularity and matrix GAG content, the macroscopic appearance and the percentage of cartilage under the tidemark. Biophysical stimulation is a non-invasive therapy, free from side effects and should be started soon after BMC transplantation to increase the quality of the regenerated tissue. However, because this is the first explorative study on the combination of a biological and a biophysical treatment for osteochondral regeneration, future preclinical and clinical research should focus on this topic to explore mechanisms of action and the correct clinical translation.

## Abbreviations

TE: Tissue engineering
MSC: Mesenchymal stem cell
ECM: Extracellular matrix
BMC: Bone marrow concentrate
PEMFs: Pulsed electromagnetic fields
ROI: Region of interest
GAG: Glycosaminoglycan.
